# Diagnostic Dilemma of Thrombotic Microangiopathy in Pregnancy

**DOI:** 10.1016/j.ekir.2020.10.036

**Published:** 2020-11-13

**Authors:** Kati Kaartinen, Leena Martola, Sari Aaltonen, Seppo Meri

**Affiliations:** 1 Department of Nephrology, Helsinki University Central Hospital, University of Helsinki, Helsinki, Finland; 2 Department of Bacteriology and Immunology, Helsinki University Central Hospital, University of Helsinki, Helsinki, Finland

Preeclampsia and hemolysis, elevated liver enzymes, and low platelet count in preeclampsia (HELLP) syndrome are characterized by new-onset hypertension, proteinuria, acute kidney failure, elevated liver enzyme levels, and low platelet counts as well as fragmentation hemolysis. Preeclampsia affects 3% to 6% and HELLP syndrome affects 1% to 2% of all pregnancies, causing significant morbidity for both the mother and the fetus.^1^ Some controversy exists as to whether HELLP syndrome is a severe form of preeclampsia or is a separate disorder.^2^^,^^3^ The cellular and molecular mechanisms underlying preeclampsia and HELLP syndrome are still unclear, but growing evidence suggests that placental clearance problems, complement dysfunction, and endothelial injury contribute to their pathogenesis.^4^^,^^5^ Whether complement activation is part of the pathogenesis of preeclampsia or a response to placental ischemia or injury requires further investigation.^3^^,^^6^ Rare mutations in genes encoding proteins regulating the alternative pathway of the complement have been reported in up to 50% of patients with HELLP syndrome, implying it is a disease of the alternative pathway of the complement.^1^ This is supported by the fact that the HELLP syndrome has features of thrombotic microangiopathy (TMA).^7^

Atypical hemolytic uremic syndrome (aHUS) is a rare genetic disorder caused by a misdirected attack of the alternative complement pathway manifesting as TMA.^8^^,^^9^ Several—mostly heterozygotic—mutations have been discovered but the penetrance of the disease is incomplete. Mutations can be found in only 50% to 70% of patients with clinical diagnoses of aHUS, making it a diagnosis of exclusion.S1,S2 In a small proportion of patients, aHUS is caused by autoantibodies against the soluble inhibitor factor H.S1 A trigger to activate complement is needed to manifest the disease, and pregnancy is a powerful trigger. In 20% of women with aHUS, the diagnosis is made during pregnancy or within 6 weeks postpartum with a preponderance of postpartum diagnoses.^1^^,^S3,S4 Antenatal aHUS often leads to adverse outcomes (prematurity, fetal growth restriction, miscarriage, renal insufficiency, or new-onset hypertension).^1^^,^S5 The diagnosis of aHUS is suggested by the presence of microangiopathic hemolytic anemia, thrombocytopenia, and renal failure in a patient with normal disintegrin and metalloproteinase with a thrombospondin type 1 motif, member 13 (ADAMTS13) activity, and a negative Shiga toxin test result.S4

We describe a case of differentiating between preeclampsia or HELLP syndrome and aHUS in a pregnant patient. She had a history of pregnancy-related hypertensive disorders in previous pregnancies, miscarriages, and subsequent chronic kidney disease culminating in aHUS in the most recent pregnancy. Subsequently, 2 complement C3 gene variants and a deficiency of factor H–related proteins 1 and 3 (FHR1, FHR3) were discovered. Evolution of the disease was indicative of aHUS rather than of a mere pregnancy-related disorder.

## Case Presentation

A 31-year-old woman of Middle Eastern descent was referred to the Department of Nephrology in Helsinki University Hospital in May 2018. She had had 2 miscarriages in 2012 and 2014 and 2 successful pregnancies in 2013 and 2015 (vaginal deliveries at weeks 39 and 36, respectively), proteinuria, and rising blood pressure in both pregnancies. However, only 1 slightly elevated (99 μmol/l) creatinine value was previously obtained in 2012. Medical records from another hospital revealed that mild reduction of platelets and anemia were evident at the time of delivery in 2015 and that she had had anemia throughout the pregnancy. Her uncle had renal impairment of unknown origin.

At the time of presentation at gestational week 27, the estimated glomerular filtration rate was 21 ml/min per 1.7 3 m^2^ and 24-h protein excretion was 4.5 g/d. Renal ultrasonographic images revealed a horseshoe kidney. Labetalol was administered 2 weeks earlier for *de novo* hypertension, but her blood pressure remained still elevated. At 29 weeks of pregnancy, the patient was admitted because of headache, rising creatinine levels, and elevated blood pressure. Because of progressive uremia and imminent preeclampsia or HELLP syndrome, a cesarean section was performed the next day. At this point, partial HELLP syndrome according to the Tennessee classification system was evident. The evolution of various laboratory values is shown in Table 1.

**Table 1:** Laboratory variables at different time points

Laboratory variable, unit (reference values)	First contact with primary care (1/2018)	First contact with university hospital (5/2018)	Delivery (6/2018)	Delivery + 3 days	Discharge, delivery + 12 days	Last outpatient visit (1/2020)
**Blood cell count**						
Hemoglobin, g/l (117–155)	105	105	98	85	82	109
B-Trom 150-360 E9/l (150–360)	201	210	148	57	306	238
**Renal variables**						
Plasma creatinine, μmol/l (50-90)		2.31	3.96	3.68	3.3	4.86
eGFRepi, ml/min/1.73 m^2^ (≥90)		27	14	16	18	11
Urine albumin/creatinine ratio, mg/mmol (<3.5)		227.8	460.3	302.8	491	49.2
Urine hematuria E6/l (<20), n		28	17		32	4
**Hemolytic variables**						
Plasma LDH, U/l (115-235)			389	399	255	141
Plasma haptoglobin, g/l (0.29-2)			1.24	< 0.1	1.7	1.19
Blood schistocytes, % (<1%)				1.1	1.1	0
Erythrocyte direct Coombs-test (positive/negative)						Negative
**Complement and other variables**						
Plasma C3, g/l (0.65-1.32)					2.16	
Plasma C3d, U/ml (<7)					3.9	
Plasma factor B, g/l (0.1-0.4)					0.30	
Plasma factor Bb activation product, μg/ml (<4)					0.7	
Plasma C4, g/l (0.11-0.32)					0.71	
Plasma C4d μg/ml (<7)					6.6	
Complement 3 nephritic factor (negative/positive)					Negative	
Plasma SC5b-9, ng/ml (<366)					423	
Plasma ADAMTS13, % (40-130%)					57	
**Liver function variables**						
Plasma ALT, U/l (<35)			11	140	39	<9
Plasma ALP, U/l (35-105)					115	76
**Blood pressure, mm Hg)**		155/95	159/102	170/90	135/91	118/77

ADAMTS13, disintegrin and metalloproteinase with a thrombospondin type 1 motif, member 13; ALT = alanine aminotransferase; ALP, alkaline phosphatase; AST, aspartate aminotransferase; C3, complement 3; C4, complement 4; C3nef, complement 3 nephritic factor; C3d, complement 3 activation product; C4d, complement 4 activation product; eGFRepi, estimated glomerular filtration rate, calculated according to the Chronic Kidney Disease Epidemiology Collaboration (CKD-EPI) equation; FacB, factor B; FacBb, factor B activation product; LDH, lactate dehydrogenase; SC5b-9, membrane attack complex.

Soon after delivery TMA emerged. Clotting panel and differential diagnostic testing revealed no abnormalities. Renal biopsy was deemed impossible because of the renal anomaly. Some postpartum improvement occurred in renal function, and it was somewhat unclear to which extent unknown chronic renal disease combined with HELLP syndrome explained the changes in various laboratory values. At this point, aHUS was not deemed likely; therefore, complement medication was not started. The patient then progressed to end-stage renal disease during follow-up and is currently awaiting kidney transplantation.

Complement gene investigations were performed by exome sequence and copy number variation analyses, including *ADAMTS13, C3, CD46, CFB, CFH, CFHR5, CFI, DGKE,* and *THBD* genes (Hemolytic Uremic Syndrome Panel, version 4; Blueprint Genetics Oy, Helsinki, Finland).

Two heterozygous missense variants in C3 were found: c.3697A>C, p.(Thr1233Pro) and c.1027C>T, p.(Arg343Cys). To our knowledge, there have been no previous publications or reports of the Thr1233Pro variant in the disease-related variant databases such as ClinVar (National Center for Biotechnology Information, Rockville, MD) or the Human Gene Mutation Database (Cardiff University, Cardiff, Wales, UK). It also has not been observed in the reference population cohorts of the Genome Aggregation Database (gnomAD) (a coalition of investigators, Broad Institute, Cambridge, MA) covering >120,000 exomes and >15,000 genomes. All *in silico* tools (polymorphism phenotyping [PolyPhen], Harvard University, Cambridge, MA; sorting intolerant from tolerant [SIFT]; and MutationTaster [MutTaster]) predict the substitution as deleterious. The second variant, Arg343Cys, has been observed in 13 individuals heterozygous for this variant in the SIFT database but no homozygotes have been observed. To our knowledge, this variant has not been described or reported in the disease-related variant databases (ClinVar or Human Gene Mutation Database). The variant is predicted tolerated by SIFT and MutTaster, and probably damaging by polymorphism phenotyping (PolyPhen).

Immunoblotting analysis of the patient’s serum using a polyclonal anti–factor H antibody showed that she lacked FHR1-protein (both α and β chains), suggesting that she had a homozygous deletion of the C*FHR1-3* gene region (Jackson ImmunoResearch, Cambridgeshire, UK and Agilent Dako, Santa Clara, CA). However, no autoantibodies against factor H were detected in an enzyme-linked immunosorbent assay (standardized in-house ELISA assay). A standardized in-house enzyme-linked immunosorbent assay was used. Maxisorp plates (NUNC, Thermo Fisher Scientific, Vantaa, Finland) were coated o/n at 4 °C with human factor H (Complement Technology, Tyler, TX). After incubation with varying dilutions of patients serum and washes, the plates were treated with a goat–anti-human IgG antibody. After washing a horseradish peroxidase–conjugated donkey, anti-goat IgG is used for detection (Calbiochem; manufactured by Calbiochem, distributed by Merck, Darmstadt, Germany).

Blood complement variables obtained 12 days after the delivery showed elevated levels of C3 and C4 and a slightly increased level of the soluble terminal complement complex (P-SC5b-9), as tested by standardized enzyme-linked immunosorbent assay. C3 protein appeared abundant in the patient’s plasma, but the molecular masses of the α and β chains did not differ from control samples in immunoblotting. No higher-molecular-weight C3-related bands were detected, indicating absence of covalent C3b complexes with other proteins. Finally, the family history of renal disease and occurrence of chronic kidney disease both point to the direction of aHUS rather than merely a pregnancy-related hypertensive disorder in our patient.

## Discussion

The differential diagnosis between pregnancy-aHUS and preeclampsia or HELLP syndrome can be challenging. The diseases may also overlap, which makes diagnosis even more laborious (Table 2). Thirty percent of first-episode pregnancy aHUS patients and 25% of patients with known aHUS also had preeclampsia or HELLP syndrome as a preceding obstetric complication in a systematic review of 48 reports.S4

**Table 2:** Teaching points

• The differential diagnosis between pregnancy aHUS and preeclampsia or HELLP syndrome can be challenging. The diseases may also overlap.
• Rare mutations in genes encoding proteins regulating the alternative pathway of the complement have been reported in patients with HELLP syndrome, implying it as a disease of the alternative pathway of the complement.
• The postpartum presentation, a family history of TMA or renal disease of unknown origin, and persistence of thrombotic microangiopathy after delivery favor the diagnosis of aHUS over preeclampsia or HELLP syndrome.
• There is a significant risk of relapse in future pregnancies or during other complement-amplifying conditions in both. Complement blockade is the treatment of choice for aHUS, whereas the treatment of choice for pregnancy-related hypertensive disorders is termination of pregnancy.

aHUS, atypical hemolytic uremic syndrome; HELLP, hemolysis, elevated liver enzymes and low platelets; TMA, thrombotic microangiopathy.

Clinical clues may be of valuable assistance because results of complement analyses, except for ADAMTS13 activity, are virtually never available at the time of treatment decisions. The postpartum presentation, a family history of TMA or renal disease of unknown origin, and persistence after delivery favor the diagnosis of aHUS over preeclampsia or HELLP syndrome.^1^ Patients with mere pregnancy-related disorders should recover after delivery; however, the time needed for the clinical improvement in preeclampsia or HELLP syndrome is not well defined. Persistence of symptoms for more than 48 to 72 hours after the delivery is highly indicative for aHUS (Table 3).S6,S7 HELLP syndrome causes acute renal failure but it usually resolves completely, unlike acute renal failure in aHUS that requires either anti-complement treatment or plasma therapy in most cases.S5,S8,S9 In the precomplement medication era, the majority of patients with aHUS progressed to chronic dialysis or died within 3 years.^9^ An association with cesarean section with pregnancy-associated aHUS was reported with an excellent renal response to eculizumab independent of whether complement abnormalities were carried or not inherited.S10 Whether anticomplement medication would have prevented our patient’s progress toward end-stage renal disease remains unanswered.

**Table 3:** Differential diagnostics of thrombotic microangiopathies during pregnancy

Condition	Symptoms and findings	Diagnosis and helpful clues	Main treatment
HELLP or preeclampsia	MAHA, epigastric or right upper quadrant pain, nausea, vomiting, headache, visual disturbances, hypertension, acute renal failure	Usual timing after 20th gestational week, should resolve within 48–72 h after delivery	Delivery, supportive care
TTP	MAHA, fever, confusion, various predominant neurologic symptoms, and usually mild acute renal failure	Peak timing during second trimester, but can occur at any time. Plasma ADAMTS13 activity <10% due to antibody or inherited deficiency	Plasma exchange and immunosuppression (if antibody-mediated)
Acute fatty liver of pregnancy	MAHA possible, nausea, vomiting, abdominal pain, malaise, headache, jaundice, acute liver and renal failure	Usual timing after 30th gestational week, suspect if hypoglycemia, elevated ammonia, coagulopathy	Delivery, supportive care
Atypical hemolytic uremic syndrome	MAHA, various extrarenal symptoms related to multiple organs (abdominal pain, headache, altered mental status, pancreatitis, abnormal liver tests, etc.), acute renal failure	Usual timing postpartum. Diagnosis of exclusion; complement investigations may clarify the diagnosis in the long run	Eculizumab or ravulizumab (plasma exchange until TTP ruled out) and immunosuppression if factor H antibody–mediated
Secondary hemolytic uremic syndrome	MAHA, various extrarenal symptoms, acute renal failure	Diagnosis of exclusion	Depends on the cause of the disease, treatment is aimed at causative agent or condition
Shiga toxin–producing *E. coli*–associated HUS	MAHA, bloody diarrhea, fever, nausea, vomiting, abdominal pain and possibly various extrarenal symptoms, acute renal failure	Stool culture for STEC-HUS; Shiga toxin enzyme immunoassay or PCR	Supportive care

ADAMTS13, a disintegrin and metalloproteinase with a thrombospondin type 1 motif, member 13; HELLP, hemolysis, elevated liver enzymes, low platelet count; MAHA, microangiopathic hemolytic anemia; PCR, polymerase chain reaction; STEC-HUS, Shiga toxin–producing *Escherichia coli*–associated hemolytic uremic syndrome; TTP, thrombotic thrombocytopenic purpura.

Renal biopsy could provide additional information but is rarely performed. Thrombi in the microvasculature, endothelial cell swelling, accumulation of protein, and cell debris occur subendothelially in aHUS.S11 In preeclampsia or HELLP syndrome the benefits of renal biopsy data are limited, but acute tubular necrosis and endotheliosis are most often reported, although features compatible with TMA may also occasionally exist.S12–S14

Heterozygous pathogenic variants and mutations in C3 increase the susceptibility to aHUS.S1 Similarly, C3 mutations were found in patients with HELLP syndrome and partial HELLP syndrome. One patient with HELLP syndrome presented with homozygous deletion of *CFHR1* and *CFHR3*, but no information of factor H antibody was given.S15 A small subset of patients with aHUS present with a more chronic evolution of kidney disease rather than an acute presentation of aHUS. This has been observed, for instance, in the context of certain C3 gain-of-function mutations.S16 This also could have occurred in our patient, although it is difficult to prove because creatinine values were not systematically followed. Plasma complement analyses may be within normal limits even during acute phases of aHUS.^7^^,^S1 In our patient, the C3 level was not decreased, but the SC5b-9 level was slightly elevated, in line with aHUS-type misdirected complement activation. The modified Ham test or tests using endothelial cells have been suggested to help in the diagnosis, but they are available mostly for research purposes and are not yet adequately validated for clinical use.S15,S17

The impact of the 2 C3 variants on the protein function is currently not known. The mutations were located in the C3d (thioester domain, Thr1233Pro) and MG4 (Arg343Cys) domains of C3 in regions that do not directly affect complement activation (factor B binding) or inhibition (factor H). Apparently, they did not reduce C3 protein synthesis or blood levels or influence the C3 polypeptide composition. Whether they influence the 3-dimensional structure of C3 or any of its activation fragments is not known. The Arg343Cys variant introduces an additional cysteine, which could lead to abnormal cysteine pairing within the C3 protein. Binding to external ligands was excluded, because neither C3 nor C3b dimers or complexes were detected by immunoblotting. We hypothesize that either of the 2 or both mutations could affect the binding of the C3 activation product iC3b to its complement receptor 3 (CD11b/CD18) or the complement receptor 4 (CD11c/CD18), or both receptors on neutrophils, macrophages, and dendritic cells. The precise binding sites of these receptors on the iC3b fragment still remain to be investigated. In this scenario, either of the 2 C3 variants could lead to an insufficient clearance of placental debris because of abnormal opsonophagocytosis via the complement 3 and complement 4 receptors.

Our patient was also deficient in *FHR1* and *FHR3*. The underlying homozygous deletion of *CFHR1* and *CFHR3* genes is relatively common and often found (>80%) in patients with aHUS who have developed antibodies against factor H.S18 Because no factor H autoantibodies were detected, we consider this deletion irrelevant for the disease pathogenesis. However, because factor H–related proteins influence complement regulation by factor H, an effect cannot be totally excluded.

If aHUS and preeclampsia or HELLP share similar pathologic features, why even bother to differentiate these 2 conditions? Both conditions pose a significant risk of relapse in future pregnancies or during other complement-amplifying conditions. Genetic analyses and counseling are recommended in patients with aHUS.S5,S9 Complement blockade with eculizumab or ravulizumab is the treatment of choice for aHUS, whereas the treatment of choice for pregnancy-related hypertensive disorders is termination of pregnancy. However, eculizumab has also shown promising results in treating severe preeclampsia or HELLP syndrome.S19 Thus, anticomplement therapy may well become a treatment option for patients with the most severe form of preeclampsia or HELLP syndrome when termination of pregnancy is insufficient.

## Disclosures

All the authors declared no competing interests.
