# Supplementary material for: Diagnostic Dilemma of Thrombotic Microangiopathy in Pregnancy
Source: Kidney Int Rep. 2020 Nov 13;6(2):529–33. doi: 10.1016/j.ekir.2020.10.036 (PMC7879120; doi:10.1016/j.ekir.2020.10.036)
Supplement: Supplementary File (PDF) [file mmc1.pdf]

Supplementary references

S1. Kavanagh D, Goodship TH, Richards A. Atypical hemolytic uremic syndrome. Semin Nephrol. 2013, 33:508–530. doi: 10.1016/j.semnephrol.2013.08.003.

S2. Loirat C, Fakhouri F, Ariceta G, et al.; HUS International. An international consensus approach to the management of atypical hemolytic uremic syndrome in children. Pediatr Nephrol. 2016;1:15-39. doi: 10.1007/s00467-015-3076-8.

S3. Fakhouri F , Roumenina L, Provot F, et al. Pregnancy-associated hemolytic uremic syndrome revisited in the era of complement gene mutations. J Am Soc Nephrol. 2010;5:859-67. doi: 10.1681/ASN.2009070706.

S4. Gupta M, Govindappagari S, Burwick R M. Pregnancy-Associated Atypical Hemolytic Uremic Syndrome: A Systematic Review. Obstet Gynecol. 2020;135(1):46-58. doi: 10.1097/AOG.0000000000003554.

S5. Bruel A, Kavanagh D, Noris M , et al. Hemolytic Uremic Syndrome in Pregnancy and Postpartum. Clin J Am Soc Nephrol. 2017;12(8):1237-1247. doi: 10.2215/CJN.00280117.

S6. Gupta M, Feinberg BB, Burwick R M. Thrombotic microangiopathies of pregnancy: Differential diagnosis. Pregnancy Hypertens. 2018;12:29-34. doi: 10.1016/j.preghy.2018.02.007.

S7. Pourrat O, Coudroy R, Pierre F. Differentiation between severe HELLP syndrome and thrombotic microangiopathy, thrombotic thrombocytopenic purpura and other imitators. Eur J Obstet Gynecol Reprod Biol. 2015;189:68-72. doi: 10.1016/j.ejogrb.2015.03.017.

S8. Abraham *K A,* Connolly *G,* Farrell J, et al. The HELLP syndrome, a prospective study. Ren. Fail. 2001; 23: 705-713. doi: 10.1081/jdi-100107367.

S9. Fakhouri F. Pregnancy-related thrombotic microangiopathies: Clues from complement biology. Transfus Apher Sci. 2016;54(2):199-202. doi: 10.1016/j.transci.2016.04.009.

S10. Huerta A, Arjona E , Portoles J et al. A retrospective study of pregnancy-associated atypical hemolytic uremic syndrome. Kidney Int. 2018 Feb;93(2):450-459. doi: 10.1016/j.kint.2017.06.022.

S11. Goodship TH, Cook HT, Fakhouri F, et al ; Conference Participants. Atypical hemolytic uremic syndrome and C3 glomerulopathy: conclusions from a "Kidney Disease: Improving Global Outcomes" (KDIGO) Controversies Conference. Kidney Int. 2017;91(3):539-551. doi: 10.1016/j.kint.2016.10.005.

S12. Packham D, Mathews D, Fairley K, et al.. Morphometric analysis of pre-eclampsia in women biopsied in pregnancy and post-partum. Kidney Int. 1988;34(5):704-11. doi: 10.1038/ki.1988.236.

S13. Hecht J, Jaume Ordi J, Carrilho C, et al. The pathology of eclampsia: An autopsy series. Hypertens Pregnancy 2017;36(3):259-268. doi: 10.1080/10641955.2017.1329430.

S14. Ye W, Shu H, Wen Y, et al. Renal histopathology of prolonged acute kidney injury in HELLP syndrome: a case series and literature review. Int Urol Nephrol. 2019;51(6):987-994. doi: 10.1007/s11255-019-02135-z.

S15. Vaught AJ, Braunstein EM, Jasem J, et al. Germline mutations in the alternative pathway of complement predispose to HELLP syndrome. JCI Insight. 2018;3(6):e99128. doi: 10.1172/jci.insight.99128.

S16. Timmermans S, Abdul-Hamid MH, van Paassen P; Limburg Renal Registry. Chronic thrombotic microangiopathy in patients with a C3 gain of function protein. Nephrol Dial Transplant. 2020;35(8):1449-1451. doi: 10.1093/ndt/gfaa050.

S17. Noris M, Galbusera M, Gastoldi S, et al. Dynamics of complement activation in aHUS and how to monitor eculizumab therapy. Blood. 2014;124(11):1715-26. doi: 10.1182/blood-2014-02-558296.

S18. Dragon-Durey MA, Sethi S, Bagga A, et al. Clinical Features of Anti-Factor H Autoantibody–Associated Hemolytic Uremic Syndrome. J Am Soc Nephrol. 2010; 21(12): 2180–2187. doi: 10.1681/ASN.2010030315.

S19. Burwick RM, Feinberg BB. Eculizumab for the treatment of preeclampsia/HELLP syndrome. Placenta. 2013;34(2):201-203. doi: 10.1016/j.placenta.2012.11.014.
